# Distal Femoral Focal Deficiency

**DOI:** 10.5435/JAAOSGlobal-D-22-00091

**Published:** 2022-09-02

**Authors:** Venla Soini, Mikko Haara, Arimatias Raitio, Johanna Syvänen

**Affiliations:** From the Department of Paediatric Surgery and Paediatric Orthopaedic Surgery, University of Turku and Turku University Hospital, Turku, Finland (Dr. Soini, Dr. Raitio, Dr. Syvänen), and the Department of Paediatric Orthopaedic Surgery, Helsinki University Hospital, Helsinki, Finland (Dr. Haara).

## Abstract

Distal femoral focal deficiency is an extremely rare type of congenital femoral deficiency that comprises hypoplasia of the distal femur, with a normally developed hip. We represent a unique case of distal femoral hypoplasia and deficiency of knee extensors, childhood follow-up and final treatment with exarticulation, and a comparison with previous literature.

Congenital femoral deficiency (CFD) is a rare congenital anomaly with a reported incidence of 1:50,000 births.^1-3^ CFD consists all the femoral deficiencies from mild focal hypoplasia to severe deformity.^4^ Most of the patients with CFD have proximal focal femoral deficiency (PFFD). Distal femoral focal deficiency (DFFD) is a type of deficiency that encompasses hypoplasia of the distal femur, with a normally developed hip. To the best of the authors’ knowledge, it has been described in English literature in only three case reports previously.^5-7^

Distal femoral focal deficiencies have been previously represented in Pappas and Paley classification schemes for congenital femoral deficiencies.^3,8^ Distal deficiencies are classified as Pappas type IX and/or Paley type 4. Taylor et al. also proposed a separate classification scheme for DFFD, presenting types A, B, and C based on the Aitken^9^ classification. Type A has an irregular distal femoral epiphysis with a bony connection between femoral components. The femur may be shortened. In type B, the distal epiphysis is present, but there is no osseous connection between the diaphysis and the distal epiphysis. In addition, the femoral length is shortened. In type C deficiency, the epiphysis is absent and the femoral segment is shortened (Figure 1).^7^ We represent a case of DFFD and combined deficiency of anterior tight muscles and comparison with the previous reports.

**Figure 1:**
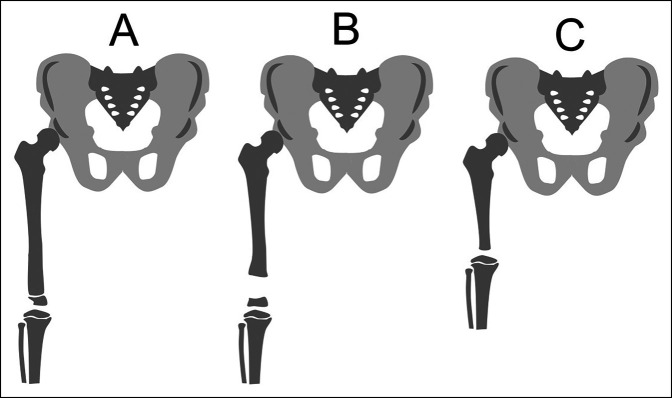
Diagram showing the classification of distal focal femoral deficiency.

## Case Report

A male infant was born with no family history of limb deficiencies. An elective section was done because of breech presentation.

The baby was referred to pediatric orthopaedics at the age of 6 weeks. Clinically, the right femur was approximately 2 cm shorter than the left one. In addition, there was an extension deficit of 90° in the knee joint, and abduction of the right hip was limited to 60° compared with 90° on the left side. The radiographs of the spine and the ultrasonography of hips, heart, and abdomen were normal, and the right femur was 1.6 cm shorter. The patient was primarily diagnosed with PFFD, Aitken type A.

At the age of 4 months, the extension of the knee was limited to 60°, and serial casting was initialized. After a couple of casting rounds, the treatment was discontinued because the family wanted to try other treatment options.

At the age of 10 months, treatment was continued with bilateral adductor tenotomy and botulinum toxin injections to anterior thigh muscles. Serial casting was also reinitialized.

**Figure 2:**
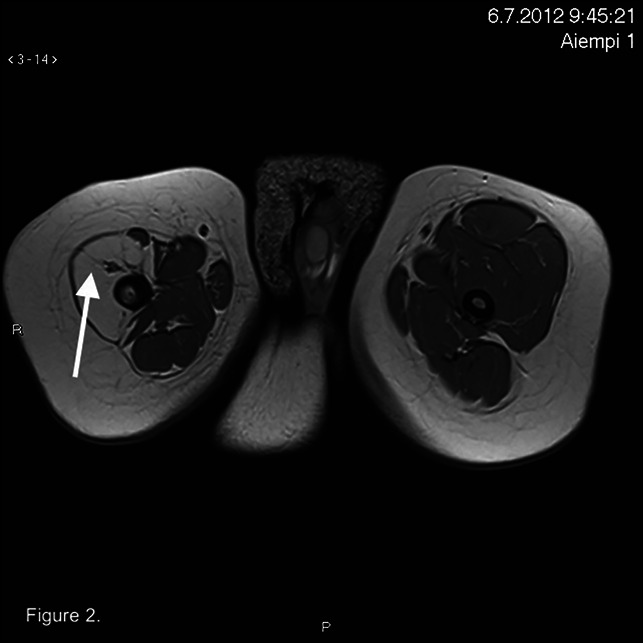
MRI image showing an arrow pointing to the missing quadriceps femoris muscle.

At the age of 1 year, the extension defect after casting was minimal. In the MRI scan, there was an absence of the quadriceps femoris muscle (Figure 2). Collateral and cruciate ligaments and posterior thigh muscles were normal. The patient learned to walk at the age of 1.5 years and was an active toddler.

Night-time orthosis treatment was used until the age of 4 years. In clinical examination, there was a 2.5- to 3-cm limb discrepancy, and the knee condyles palpated asymmetrically. Another round of series casting was initialized because of increased extension deficit. Regardless, the range of motion was limited to only 30° to 60° 1 year later.

**Figure 3:**
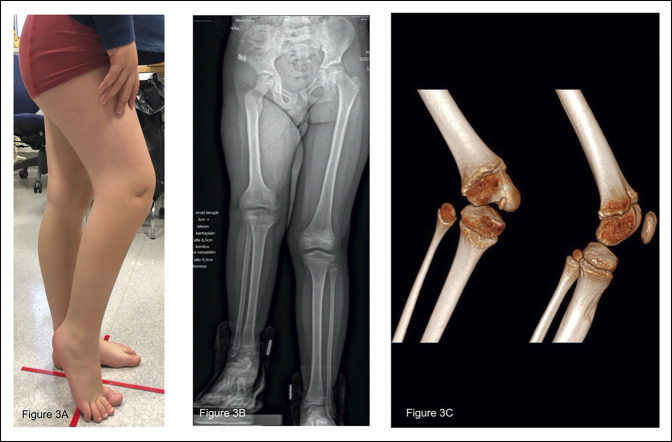
**A**, Photograph showing the clinical situation at the age of 7 years. **B**, Long lower limb radiograph at the age of 8 years showing 6.9-cm anatomical limb-length discrepancy. **C**, 3D computer tomography image showing the bony anatomy in the knee.

When inspected at school age, the pelvis was symmetrical with an 8-cm additional plate under the right foot. Discrepancy was caused by femoral hypoplasia and flexion contracture of both knee and hip joints. The expected limb-length discrepancy in adulthood was calculated to be 6.1 cm and 1.6 cm in the femur and tibia, respectively. The patient was walking with an elevated heel as presented in Figure 3, A. Radiographically, the patient's right distal femoral epiphysis was severely deformed and hypoplastic with an almost missing patella. The lateral condyle is notably small and lacking the distal most part of the condyle. The physis itself seems almost normal looking. The medial side is not symmetric to the left counterpart either but only has a mildly deformed, seemingly normal-sized medial condyle. (Figure 3, C).

Owing to functional disability, several treatment options were considered. The initial plan was first to straighten the knee and subsequently lengthen the limb. Extension osteotomy of the femur with tibial slope correction accompanied with an external circular fixator to gradually extend the knee joint was primarily recommended. Other options included rotationplasty and amputation. After careful consideration and discussion with the family, an extra-articulation of the knee was done at the age of 10 years. The patient has now been followed up after the surgery and is adapting well to his new prosthesis six months postoperatively.

## Discussion

We have presented a unique case of DFFD with deficit of the extensor apparatus of the knee. To the best of the authors' knowledge, there are no previous reports of this kind of femoral anomaly.

Previously published cases by Gilsanz and Taylor et al^7^ have been described as type C. According to our interpretation, the case presented by Tsou^5^ could be graded as type B because there was no bony connection in the presented radiograph between the femoral diaphysis and the distal epiphysis. Our patient's deficiency could be graded as DFFD type A because there is an irregular distal epiphysis with a bony connection. In addition, the femur was shortened. Taylor et al stated that their patient was originally incorrectly diagnosed as PFFD and only later diagnosed as DFFD. They speculated that there could be other misdiagnosed patients as well. Similarly, our patient was first diagnosed as PFFD, Aitken type A, at the age of 6 weeks.

The reported treatment methods have varied from patient to patient. Tsou et al described a planned lengthening operation, but the results remain unclear. Gilsanz conducted an exarticulation of the knee, whereas Taylor et al fitted a prosthesis evidently without operation.^6,7^ In both cases, the limb-length discrepancy was markedly bigger than in our patient. Therefore, treatment was required markedly earlier than in our case.

Rotationplasty is an old and well-recognized treatment method for severe CFD and lower limb malignancies.^10,11^ There are many complications associated with this technique including skin flap necrosis with an incidence of over 50% in former studies.^12-14^ Unlike most patients with CFD, our patient had an almost normally functioning hip. Therefore, rotationplasty would have decreased the range of motion of the hip joint. In addition, the family was worried about the psychological burden of the unusual appearance of the limb after rotationplasty, although published literature does not support these concerns.^15,16^

Although reconstructive procedures were initially considered, amputation was chosen for several reasons. First, the range of motion in the knee was markedly limited and the extensor force was basically nonexistent. Second, the family did not want to have repetitive operations with first extending the knee and then lengthening the femur. Third, the parents did not want to take complication risks concerning reconstructive surgery.

Amputation was chosen after thorough discussion with the family, and it provided a permanent solution with fast recovery without the need for additional surgery. Elmherig et al summarized in their meta-analysis what other studies have shown that the later quality of life of the amputated limb deficiency patients does not markedly differ from the ones who have undergone a reconstruction.^17-19^ Our patient is very satisfied and is learning fast to walk and train with an orthosis without limb-length discrepancy and a straight pelvis and spine.
